# Major drug resistance mutations to HIV-1 protease inhibitors (PI) among patients exposed to PI class failing antiretroviral therapy in São Paulo State, Brazil

**DOI:** 10.1371/journal.pone.0223210

**Published:** 2019-10-01

**Authors:** Giselle de Faria Romero Soldi, Isadora Coutinho Ribeiro, Cintia Mayumi Ahagon, Luana Portes Ozório Coelho, Gabriela Bastos Cabral, Giselle Ibette Silva López Lopes, João Leandro de Paula Ferreira, Luís Fernando de Macedo Brígido, on behalf of the Salvage Therapy Working Group

**Affiliations:** Núcleo de Doenças de Vinculação Sanguínea ou Sexual, Laboratório de Retrovírus, Centro de Virologia, Instituto Adolfo Lutz, São Paulo, São Paulo, Brazil; Hôpital Bichat-Claude Bernard, FRANCE

## Abstract

**Background:**

Protease inhibitors (PI) are especially important in salvage therapy. Previous treatment failure with a PI containing regimen may elicit resistance mutations, reducing PI susceptibility and limiting treatment options. The aim of this study was to describe major PI mutations among patients exposed to at least one PI to evaluate predictors of mutation emergence and the impact of subtypes on resistance.

**Methodology:**

Partial HIV-1 *pol* sequences (Sanger Sequencing) from patients exposed to PI with virological failure were genotyped from January 2014 to December 2017. Drug resistance mutations (DRM), antiretroviral susceptibility (GSS) and subtypes, along clinical and laboratory parameters, were evaluated using logistic regression to access the predictors of mutation emergence.

**Results:**

In 27.5% (466/1696) of the cases at least one major PI mutations was identified, most commonly M46 (14.7%), V82 (13.8%) and I54 (13.3%). Mutations to NRTI and NNRTI were observed in 69.6% and 59.9%, respectively, of the 1696 sequences. Full activity to darunavir was predicted in 88% (1496/1696), but was only 57% among those with at least one PI-DRM. Subtype C sequences had less major PI-DRMs (10%, 9/87) compared to B (28%, 338/1216) or F (35%, 58/168) (p <0.001) but adjusted analysis suggested that this association is not independent from a shorter treatment time and fewer regimens (OR 0.59, Confidence Interval 95: 0.2–2.5, p = 0.48). Subtype F, together with NRTI mutations and longer time on treatment was associated to presence of PI-DRM, to a lower darunavir GSS and to mutations at codon I50.

**Conclusions:**

Among patients with PI-DRM, full activity to darunavir was compromised in almost half of the cases and efforts to detect failure at earlier time are warranted, particularly for HIV-1 subtype F that showed association to the emergence of resistance, with potential impact in protease inhibitors sequencing. Furthermore, NRTI mutations may serve as an indicative of sufficient adherence to allow PI-DRM emergence.

## Introduction

Information on protease inhibitor drug resistance mutations (PI-DRM) is an important component in the planning of salvage therapy. Contrary to NRTI drugs, where resistance may not be an impediment for drug recycling in second-line therapy [1–3], and to first generation NNRTI drugs, that should be avoided in exposed patients even without detectable mutations, detection of PI-DRM may help in the selection of PI drugs to be used as part of salvage regimens. Adherence reinforcement, with no regimen change, has been recommended when no mutations are detected [4].

Brazil offer free access to most ARV and older PI (Indinavir, ritonavir, nelfinavir and Saquinavir) had been used since late 90’. Although the recommended first ARV regimens in Brazil at the time of the study was efavirenz plus two NRTI, PI could be used as an alternative as first line (lopinavir or atazanavir) and at second and subsequent regimens at physician’s discretion. Darunavir (or tipranavir) however were reserved for salvage therapy of cases with documented genotypic resistance to the PI class and only occasionally dispensed due to adverse effects. Some patients using PI had to switch to other drugs due to discontinuation, as indinavir in 2013 and fosamprenavir in 2016. During the last year of this study (2017) darunavir use was also permitted as an alternative to other PI as in cases with intolerance and lopinavir use was discontinued. Integrase inhibitors (raltegravir and dolutegravir), maraviroc, efuvurtide and the new NNRTI generation etravirine were reserved for cases with extensive genotypic resistance. State committees evaluated physicians request for these newer drugs and authorized its dispensation based in patients’ history and HIV genotype. Confirmed viral load above 1,000 copies/mL after at least 6 month on treatment was the national criteria for genotyping at the time of the study, but our laboratory additionally offered genotype test for cases with persistent low level viremia, 2 or more detectable viral load after 6 month on treatment. Dolutegravir plus Tenofovir and 3TC became the recommended first line therapy in 2017.

The São Paulo State has the largest population in Brazil and currently treats over 100,000 individuals living with Human Immunodeficiency Virus (HIV) [5]. With a predominantly subtype B epidemic, it has, since the early observations in the 1990s, evidence for a minor (circa 10%) of subtype F infected individuals and BF recombinants. More recently, subtype C has been increasingly identified [6], surpassing HIV-1 F as the second subtype among recently diagnosed patients in the region [7]. Subtype C is responsible for about half of the HIV epidemic worldwide and although subtype F is rare or absent in most places, it circulates in most of South America, where BF recombinants are commonly found [8]. Apart from Romania, it is also rare in Europe, but the rapid expansion of HIV-1 subtype F among men who have sex with men (MSM) in Galicia, Spain [9–10] warrants that HIV molecular epidemiology may change swiftly.

HIV polymorphisms are a consequence of HIV diversity and non-B subtypes, by definition, have distinct nucleotide composition that allows the distinction of sequences by genetic distance. These differences have been a concern for HIV diagnostic tests, vaccine studies and may potentially impact resistance emergence and evolution. Evaluations of differential expression of drug resistance mutations (DRM) among subtypes have been provided by international collaborations [11]. However, contemporary resistance prevalence is not well known, as few observational studies have been conducted since the introduction of newer boosted protease inhibitors [12–13].

As treatment guidelines in Brazil and elsewhere do not consider HIV subtype, São Paulo State provides an interesting scenario to evaluate the resistance profile of three co-circulating subtypes (B, C and F). Treatment is initiated with no influence of HIV subtype assignment and patients are therefore exposed to initial and subsequent antiretroviral therapy (ART) regimens based solely in viral load response, tolerability issues and, where genotyping is available, resistance profile. To access predictors of major PI resistance and potential influence of HIV subtypes we evaluated PI exposed patients failing ART.

## Materials and methods

Patients were included when: a) a partial HIV-1 *pol* sequence was available at our laboratory; b) it was generated due to a request for genotyping test for virological failure of patients on antiretroviral therapy and; c) treatment history included the use of one or more PI containing regimen. Demographic and laboratory data were obtained from physician request form or from laboratory and treatment databases. Viral load quantification at the time of genotyping was obtained with Abbott Real Time HIV-1 (ABBOTT Molecular, USA) and the last CD4 T cell count (flow cytometry, FACSCalibur, BD, San José, USA) performed prior to genotype test, was considered for the analysis. The study was approved by the Institutional Ethical and Scientific Committees (Comitê de Ética e Pesquisa do Instituto Adolfo Lutz, Parecer-018/11 and Comitê Técnico Científico do Instituto Adolfo Lutz- Projeto-84D/10).

All analyzed sequences (Sanger Sequencing. Acession numbers: MN235946—MN237641) were generated from plasma RNA obtained by either commercially available TRUGENE® HIV-1 Genotyping Assay / OpenGene® DNA System kit (Siemens Healthcare Diagnostics, Tarrytown, NY) or by an “in-house” protocol [14]. Briefly RNA was extracted from 140 μL of plasma either by Qiagen (QIAamp Viral RNA Mini Kit) or Abbott M2000 automatic extractor, retrotranscribed and amplified by a one-step RT-PCR reaction using High Fidelity Taq platinum and SuperScript III, followed by Nested PCR and sequencing with Big Dye^™^ Terminator Cycle Sequencing Ready Reaction—ABI Prism® (Life Technologies, USA), resolved at an ABI Prism 3130XL Genetic Analyzer automatic sequencer (Life Technologies, USA). For samples with low viral (< 1,000 copies/mL), 2 mL of plasma were centrifuged at 30,000 RCF for 1 h 30 min at 4°C, followed by manual extraction from 140 uL.

Sequences were edited with Sequencher 4.7 software (Gene Codes) or RECall (http://pssm.cfenet.ubc.ca). For determination of the viral subtype, the sequences were analyzed by the REGA HIV-1 Subtyping Tool—Version 3.0 (http://dbpartners.stanford.edu/RegaSubtyping/), jumping profile Hidden Markov Model—jpHMM (http://jphmm.gobics.de/submission_hiv) and National Center for Biotechnology Information-NCBI (https://www.ncbi.nlm.nih.gov/projects/genotyping/formpage.cgi). Sequences with no concordance in subtype assignment as HIV-1 B, C, F or as BF or BC recombinant were classified as “other”. Mutations associated with antiretroviral resistance were determined using the Genotypic Resistance Interpretation Algorithm (GRI) Stanford HIVdb version 8.8 algorithm (Stanford db).

The genotypic susceptibility score (GSS), also performed at Stanford db, used to predict the collective impact of mutations on each drug. The results were expressed for each antiretroviral on a scale of 0 to 1, according to the predicted impact on each ARV activity. The score 0 predicts high level resistance; 0.25 intermediate resistance; 0.5 low-level resistance; 0.75 potentially low-level resistance and 1 for predicted full drug activity (no resistance). When two or more sequences from a patient were available, the first (older) sequence was used in the analysis.

The data were analyzed in Stata v8 (Stata Corp, USA). The results of the continuous variables were presented as median and interquartile range (IQR: 25^th^-75^th^). For the comparison of continuous variables between two or more groups, Mann-Whitney or Kruskal-Wallis tests, respectively, were used. Dichotomous variables were compared by Pearson chi test or Fisher’s exact test. Logistic regressions were used to evaluate independence of associations.

Continuous variables were dichotomized as; low viral load (below vs above 1,000 copies/mL) and high viral load (below vs above 100,000 copies/mL), age (lower quartile vs. three other quartiles), CD4 T cell count (below vs. above 200 and below vs. above 500 cells/mm^3^), treatment time (lower quartile vs. three higher quartiles and below vs. above the median value), number of regimens (one or two vs. more than two regimens) and GSS (score 1 vs. any other score). Dichotomization that generated a more significant association was described and selected for logistic regression analysis. Variables that presented p ≤0.2 in the unadjusted analysis were retained for the adjusted analysis. The level of statistical significance was assumed as p <0.05, two tailed.

## Results

From January 2014 to December 2017 1834 adult patients had HIV-1 polymerase (protease + partial reverse transcriptase) gene sequences generated at our laboratory to provide genotyping test for patients with virological failure. We included in this study 1696 sequences of patients from different regions of São Paulo State that had associated basic demographic data and treatment information of prescription of at least one PI containing regimen. Table 1 describes demographic and laboratory characteristics from the 1696 patients with information of PI use according to the presence or not of one or more major protease inhibitor drug resistance mutation.

**Table 1:** Demographic and Laboratory characteristics of patients at the time of genotype collection.

	All	With PI-DRM	Without PI-DRM
Number of patients	1696	466	1230
Male sex (%)	984 (58%)	281 (60.3%)	703 (57.2%)
Age (years)	43 (36–51)	46 (40–53)	42 (34–50)
Number of regimens	2 (1–4)	4 (2–5)	2 (1–3)
Time on ART (years)	8 (4–14)	13 (8–17)	6 (2–12)
CD4+T count2^1^	271 (135–467)	289 (139–494)	263 (131–457)
HIV RNA^2^	3.79 (3.02–4.65)	3.73 (2.95–4.57)	3.82 (3.04–4.69)
Viral Load <1,000c/mL	368 (24.2%)	116 (26.9%)	252 (23.2%)
Viral Load >100,000c/mL	412 (24.3%)	93 (20%)	319 (25.9%)
Subtypes (%)			
HIV-1 B	1216 (71.7%)	338 (72.5%)	878 (71.4%)
HIV-1 C	87 (5.1%)	9 (1.9%)	78 (6.3%)
HIV-1 F	168 (9.9%)	58 (12.5%)	110 (8.9%)
Recombinants BC	17 (1.0%)	3 (0.6%)	14 (1.1%)
Recombinants BF	194 (11.4%)	56 (12.0%)	138 (11.2%)
Others	14 (0.8%)	2 (0.4%)	12 (1.0%)
Mutations (%)			
NRTI	1181 (69.6%)	444 (95.3%)	737 (59.9%)
NNRTI	1016 (59.9%)	273 (58.6%)	743 (60.4%)
PI	466 (27.5%)	466 (100%)	0 (0%)

1 cells/mm^3^

2 log_10_

Results expressed as median and IQR. Mutations according to Stanford db. ART: antiretroviral therapy; NRTI: nucleotide analog reverse transcriptase inhibitor; NNRTI: non-nucleoside reverse transcriptase inhibitor; PI: protease inhibitor; DRM: drug resistance mutations; Others: sequences with subtypes other than B, C, F, BC or BF at the polymerase gene. Data related to the first sequence was used in cases with more than one sequence.

At least one nucleotide analog reverse transcriptase inhibitor (NRTI) resistance mutation was present in 69.6% (1181/1696) of the sequences, while 59.9% of them presented mutations to non-nucleoside reverse transcriptase inhibitor (NNRTI) drug class. In 27.5% at least one PI-DRM was identified. Resistance to PI was higher if only cases using a PI at genotype collection (40.5%) are considered. Among cases with viral load below 1,000 copies/mL, 31.5% had at least one IP-DRM, 27.1% for those with viral load 1,000 to 1000,000 and 24.8% for those with viral load above 100,000 (p = 0.19). Information on specific PI prescriptions was available only part of the patients (57.4%, 974/1696). Among cases with specific PI information, and considering all regimens used, lopinavir was the most prescribed PI (55.4%, 539/974), followed by atazanavir (43.5%). Older PI (saquinavir, indinavir, nelfinavir) were used by at least 28% (42/974, 102/974 and 131/974, respectively) of the cases with specific PI information. Ritonavir was used as an active PI during the early HAART era and subsequently as a booster to other PI. Drugs as tipranavir and fosamprenavir were used by less than 1% (6/974 and 3/974, respectively) of cases.

**Fig 1:**
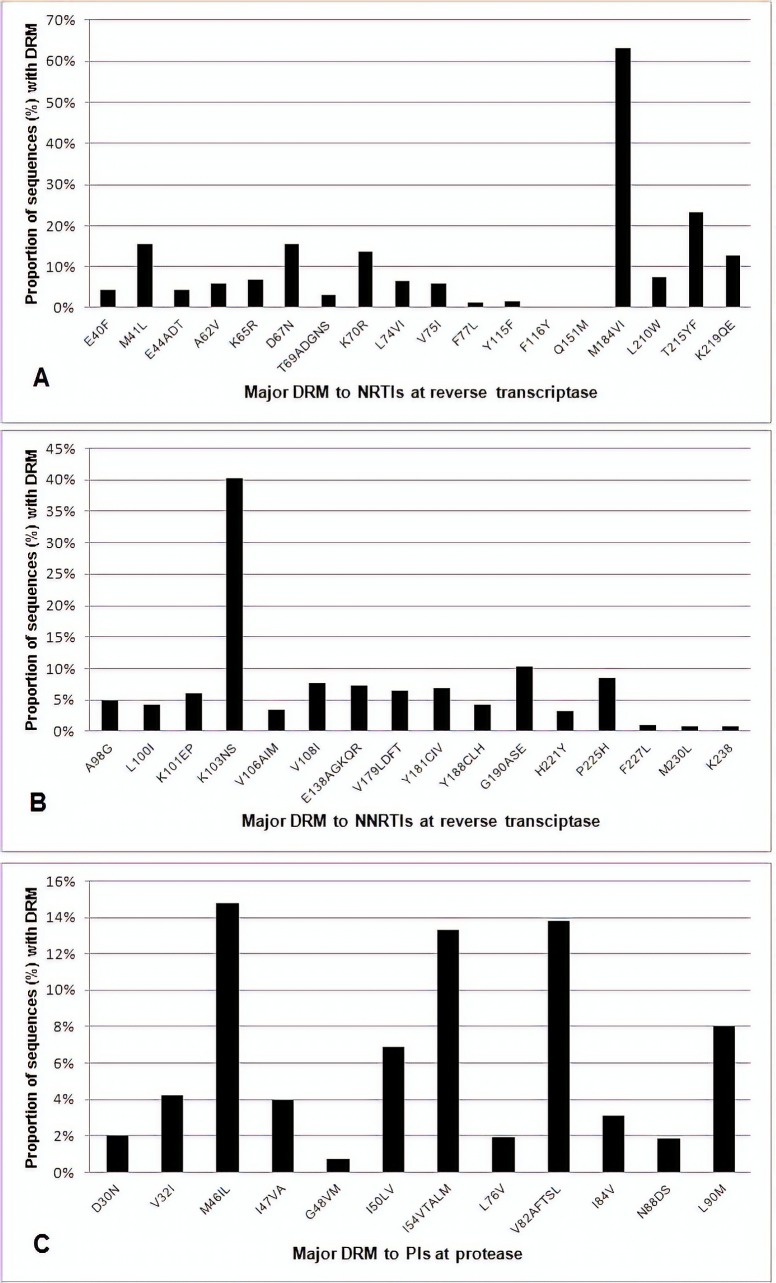
Major drug resistance mutations in patients failing therapy and exposed to at least one PI. Proportion of sequences with protease (PT) and reverse transcriptase (RT) mutations, presented by each resistance-associated codon, according to antiretroviral (ARV) class. (A) Major mutations to nucleoside RT inhibitors (NRTIs). (B) Major mutations to non-nucleoside RT inhibitors (NNRTI). (C) Major mutations to protease inhibitors (PI). Data according to the Stanford HIV Resistance Database.

Resistance at codons M184 (63%) and K103 (40.3%) were the most common mutations to the NRTI and NNRTI antiretroviral (ARV) class, respectively. The most common mutations to PI antiretroviral class were M46IL, present in 14.7% of the sequences, V82AFTSL in 13.8% and I54VTALM in 13.3%. The proportion of sequences with mutations, by resistance-associated codon, can be seen in the Fig 1.

A total of 23.8% (403/1696) sequences showed accessory resistance mutations. These accessory mutations were rare among sequences without a major PI resistance mutation (4%, 49/1230) but detected (1 to 5 accessory mutations) in most of those sequences with a major PI-DRM (76% - 354/466, p<0.001). The number of accessory mutations among cases with at least one major PI-DRM does not differ significantly across subtypes.

To better evaluate potential confounders and predictors of PI-DRM, logistic regressions analyses were performed. Table 2 depicts the unadjusted (univariable) and adjusted analysis for associations to the presence of at least one PI-DRM. Presence of a PI-DRM, at the unadjusted analysis, was strongly associated (p<0.001) to older age (>36 years), use of more than two regimens, longer time on treatment (>7 years) and with the presence of a NRTI mutation. In addition, it was also strongly associated with exposure to use of PIs as Lopinavir, Darunavir and older PIs. On the other hand, viral load (VL) levels (either proportion of cases with VL less than 1,000 or above 100,000 copies/mL) or the presence of NNRTI mutations did not show significant associations to the presence of a PI-DRM. The number of PI used by the patient was associated to a higher number of PI-DRM (p<0.001) and more than half of the patients with three or more PI prescribed had at least one PI-DRM. The adjusted analysis shows subtype F, NRTI mutations and a longer time of treatment significantly associated to the presence of at least one PI-DRM.

**Table 2:** Logistic regression to evaluate the association of demographic and laboratory variables to the presence of at least one protease inhibitor drug resistance mutation (PI-DRM).

	Unadjusted			Adjusted		
	Odds ratio	p	95% CI	Odds ratio	P	95% CI
Age above 36 Years	2.37	0.000	1.78–3.16	1.13	0.684	0.63–2.03
Male sex	0.88	0.241	0.71–1.09			
CD4 T count >200 cells/mm^3^	0.95	0.628	0.76–1.19			
CD4 T count >500 cells/mm^3^	1.21	0.142	0.94–1.57	1.21	0.484	0.71–2.04
Viral load <1,000 copies/ml	1.22	0.126	0.95–1.58	0.90	0.667	0.54–1.48
Viral load >100,000 copies/ml	0.81	0.184	0.58–1.11			
Time on treatment >7 years	4.69	0.000	3.31–6.64	1.92	0.025	1.09–3.38
Number of regimens >2	3.33	0.000	2.39–4.63	0.66	0.211	0.35–1.26
NRTI mutation	13.50	0.000	8.67–21.03	14.73	0.000	6.80–31.90
NNRTI mutation	0.93	0.494	0.75–1.15			
Exposure to IDV, SQV, NFV	4.10	0.000	3.02–5.55	4.32	0.000	2.44–7.63
LPV treatment	1.94	0.000	1.45–2.58	2.04	0.006	1.23–3.38
ATV treatment	1.38	0.023	1.05–1.82	2.05	0.005	1.24–3.37
DRV treatment	7.69	0.000	4.56–12.98	5.93	0.000	2.33–15.08
HIV-1 polymerase subtype F	1.37	0.071	0.97–1.93	2.19	0.023	1.16–4.30

Logistic regression of variables at the genotyping collection associated to PI mutations. NRTI: nucleotide analog reverse transcriptase inhibitor; NNRTI: non-nucleoside reverse transcriptase inhibitor; PI: protease inhibitor; DRM: drug resistance mutations; IDV: Indinavir; SQV: Saquinavir; NFV: Nelfinavir; LPV: Lopinavir; ATV: Atazanavir; DRV: Darunavir.

**Fig 2:**
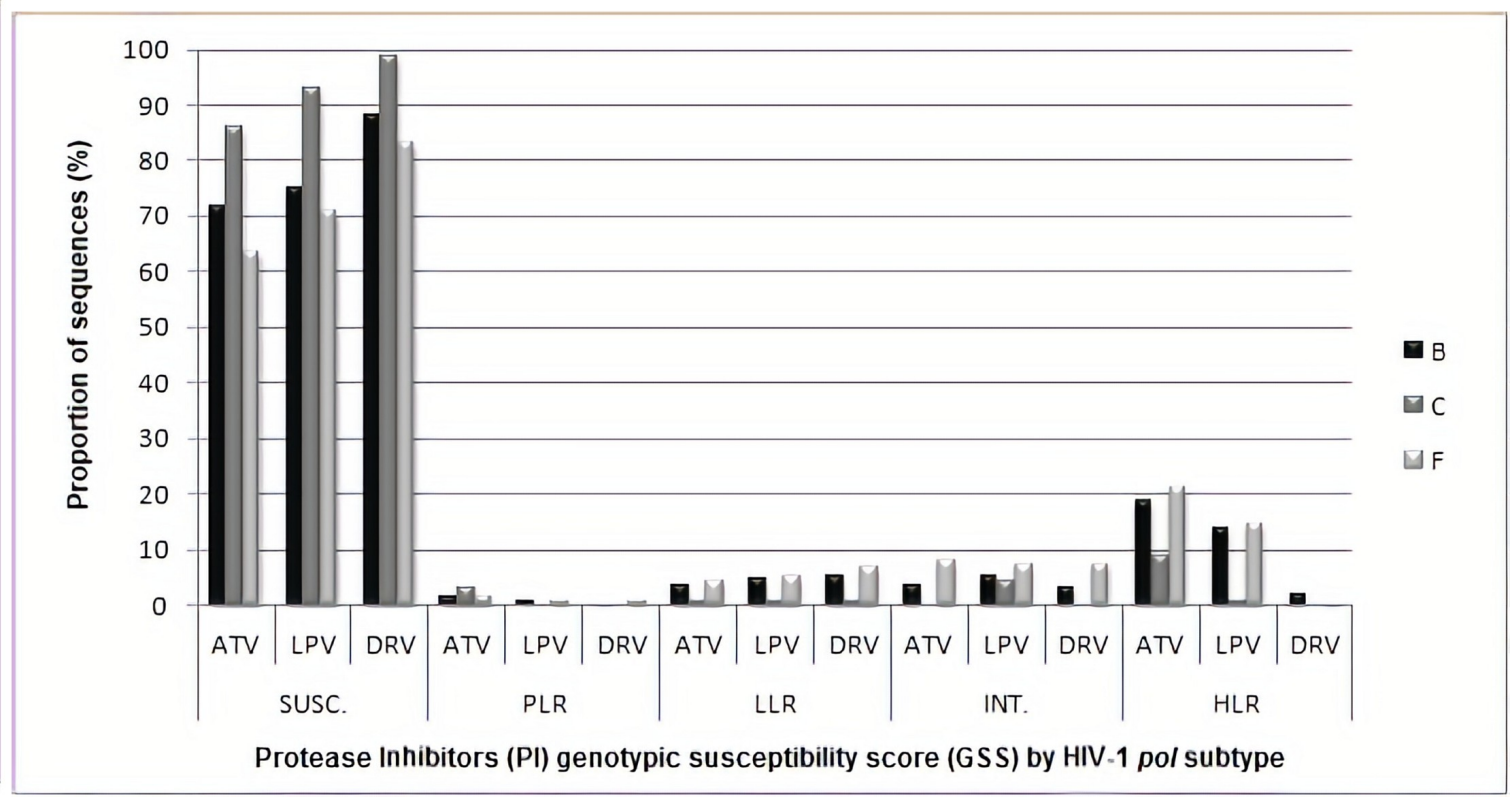
Protease inhibitors GSS and HIV-1 subtype. Proportion of HIV-1 polymerase partial sequences by subtype (B, C and F), according to the genotypic susceptibility score (GSS) to Atazanavir (ATV), Darunavir (DRV) and Lopinavir (LPV). GSS determined at Stanford HIV Resistance Database as: SUSC. for: Susceptible; PLR. for: Potential Low Resistance; LLR. for: Low-Level Resistance; INT. for: Intermediate Resistance and HLR. for: High-Level Resistance to each antiretroviral.

As shown in Fig 2, the most commonly used PIs (ATV, LPV and DRV) had full activity predicted in 71.8% (1218/1696), 75.4% (1278/1696) and 88.2% (1496/1696), respectively. However, If only cases with at least one or more PI-DRM are considered, full activity is much more limited, with 0%, 10.3% (48/466) and 57.1% (266/466), respectively. Full activity to ATV is significantly lower for subtype F (63.7%, 107/168) vs. B (71.8%, 873/1216) (p = 0.03). Proportion of full DRV or LPV activity in subtype F vs B are more comparable; DRV subtype F 83.3% (140/168) vs. 88.1%(1071/1216) in subtype B, p = 0.08; for LPV 70.8% (119/168) in subtype F vs. 74.9% (911/1216) in subtype B (p = 0.26). However, when only cases with NRTI mutations are considered, both DRV 76.1% (89/117) vs. 83.5% (726/870, p = 0.05) and ATV 50.4% (59/117) vs. 62.1% (540/870, p = 0.016) show a lower GSS for subtype F vs. B.

Logistics regressions to evaluate predictors of DRV resistance are shown in Table 3. Similarly to the presence of PI-DRM, lack of a predicted full darunavir activity is associated at unadjusted analysis to many variables, as older age, longer time on treatment, use of more regimens, some PIs and the presence of NRTI mutations, but significance is not retained at adjusted model. Subtype F, use of older PI drugs and DRV use remained significantly at adjusted analysis. NRTI mutations, which also shows a strong association to low DRV GSS score, is not included in the adjusted analysis as it predicted the outcome perfectly.

**Table 3:** Logistic regression to evaluate the association of demographic and laboratory variables to DRV susceptibility.

	Unadjusted			Adjusted		
	Odds ratio	P	95% CI	Odds ratio	P	95% CI
Age above 36 Years	2.07	0.001	1.37–3.11	1.98	0.082	0.92–4.28
Male sex	0.87	0.363	0.64–1.18			
CD4+T count >200 cells/mm^3^	1.19	0.267	0.88–1.62			
CD4+T count >500 cells/mm^3^	0.77	0.190	0.52–1.14	0.60	0.146	0.30–1.20
Viral load <1,000 copies/ml	0.83	0.332	0.57–1.21			
Viral load >100,000 copies/ml	0.91	0.638	0.60–1.37			
Time on treatment >7 years	2.70	0.000	1.70–4.30	1.11	0.769	0.60–2.24
Number of regimens >2	2.95	0.000	1.86–4.70	1.19	0.651	0.57–2.50
NRTI mutation*	13.50	0.000	8.67–2.03			
NNRTI mutation	1.11	0.520	0.82–1.50			
Exposure to IDV, SQV, NFV	3.75	0.000	2.54–5.53	3.11	0.000	1.65–5.85
LPV treatment	2.04	0.001	1.36–3.07	1.68	0.098	0.91–3.11
ATV treatment	1.06	0.777	0.72–1.55			
DRV treatment	8.17	0.000	4.96–13.47	3.95	0.000	1.83–8.54
HIV-1 polymerase subtype F	1.48	0.083	0.95–2.30	2.23	0.021	1.13–4.40

Logistic regression of variables at the genotyping collection related to darunavir (DRV) genotypic susceptibility score (GSS). NNRTI: non-nucleoside reverse transcriptase inhibitor; IDV: Indinavir; SQV: Saquinavir; NFV: Nelfinavir LPV: Lopinavir; ATV: Atazanavir.

*The NRTI resistance mutation is not included in the adjusted because it predicted perfectly success.

The evaluation of protease inhibitors resistance associated codons show that subtype C sequences had lower prevalence of PI-DRM at most resistance codons when compared to either F or B subtypes, as can be seen in Table 4.

**Table 4:** Percentage, by subtype, of protease inhibitor (PI) resistance mutations in the PI exposed group.

Amino acid in the wild virus	Location	Amino acid in the mutation presence	HIV-1 Subtype and Recombinants (%)						
			B	C	F	BC	BF	Others	Total
D	30	N	2.3	0.0	1.2	0.0	0.5	0.0	1.8
V	32	I	4.3	0.0	4.6	0.0	4.3	0.0	3.9
L	33	F	9.7	4.6	10.1	5.9	9.8	0.0	9.4
M	46	IL	14.3	1.7	16.2	14.3	14.9	6.3	13.6
I	47	VA	3.9	0.9	5.8	0.0	3.4	0.0	3.71
G	48	VM	0.8	0.0	0.6	0.0	0.5	0.0	0.7
I	50	LV	5.9	5.1	10.4	4.8	7.7	0.0	6.4
I	54	VTALM	12.8	1.7	13.3	0.0	16.8	6.3	12.4
L	76	V	1.5	0.0	2.9	0.0	3.4	6.3	1.7
V	82	AFTSL	13.0	3.4	12.1	4.8	19.8	6.3	12.9
I	84	V	3.6	0.9	1.7	0.0	1.4	0.0	2.9
N	88	S*	1.9	0.0	2.3	0.0	1.0	6.3	1.7
L	90	M	8.4	1.7	8.7	0.0	6.3	0.0	7.6

*No sequence had the amino acid D

Others: sequences with subtypes other than B, C, F, BC or BF at the polymerase gene.

Considering all major PI mutations, subtype C sequences (10.3%, 9/87) had fewer major PI mutations compared to B (27.8%, 338/1,216; p<0.001). Subtype F sequences (34.5%, 58/168) also had more mutations than C (p<0.001) but only marginally more than HIV-1 B (p = 0.07). The number of PI-DRM is also much lower in subtype C considering all sequences (p = 0.002) as well as only among those with at least one PI-DRM (p = 0.03). This may in part reflect the fact that subtype C infected patients tended to have less exposure to ARV therapy. Although total time on treatment was comparable for B and F infected patients, with 8.7 (4–14) vs. 7.0 (3–12) years, it was significantly shorter, 4.1 (1–7) years, for subtype C (p = 0.0001). Moreover, patients infected with HIV-1 C used less regimens (2, ranging from 1 to 3) than subtype B (3, 1–4) or F (2, 1–4) infected patients (p<0.01). Accordantly, adjusted analysis of subtype C along other variables show that the association is not retained (OR 0.59, Confidence Interval 95: 0.2–2.5, p = 0.48).

Although the proportion of mutations at PI resistance related codons showed a similar pattern (Table 4), that is similar rates for subtypes B and F and lower rates for subtype C, some codons, as I50, showed rates among subtype C sequences more comparable to that of B or F. Considering the specific I50L mutations to ATV, the proportion is not statistically different in HIV-1 C sequences (6.9%, 6/87 vs. B 4.4%, 76/1216, p = 0.27) overall as well as among cases with documented exposure to atazanavir (12.5%, 3/24 vs. 8.6%, 26/302, p = 0.77). However, if only cases with at least one NRTI-DRM (13.6%, 6/44 vs 6.1%, 53/870, p = 0.05) or with at least one PI-DRM (67%, 6/9vs 16%, 53/338, p<0.001) are considered, subtype C has a significantly higher prevalence of I50L mutations.

Albeit with these rates of I50L, full atazanavir activity is more commonly predicted for HIV-1 C sequences as compared to B, both overall (86.2%, 75/87 vs. 71.8%, 873/1216, p = 0.004) as well as among those with documented ATV exposure (C 87.5%, 21/24 vs. 65.2%, 197/302 in B, p = 0.03), maybe reflecting the use of alternative resistance pathways in subtype B infections. In fact, if only cases without I50L mutations are analyzed, full GSS is also higher in subtype C than in subtype B sequences (92.6%, 75/81 vs. 75.1% (873/1163), p<0.001). Similar pattern was observed for I50V (data not shown).

Mutations at I50, either Valine or Leucine, are more prevalent in subtype F then B (p = 0.03). Details on the actual PI usage are not available, but information on use to different PI, although incomplete, did not show significant differences when B and F are compared (data not shown). Nucleotide codifying the wild and resistance amino acid codons could facilitate the emergence of resistance, but subtype F does not show a clear distinction to that of B, with most sequences without DRM using the nucleotides ATT (98.7%), similar rates to that described at Stanford db “HIV-1 sequence variability tool” for subtype B in Low and Middle Income Countries (LMICs) (99.8%), B in US (98.6%), as well as for subtype C in LMICs (99.6%).

Other codons used in subtype F of this study included ATC and ATW (A or T). Mutations were also coded by similar codons observed for subtype B or C (CTT or GTT). Flanking segments are also similar, with most sequences (84.5%) showing a GGGGGA###GGAGGT motif, also the most common nucleotides observed in subtype B in North America (88.6%) or LMICs (90%), with other combinations similar to that described at Stanford db for B and C.

Table 5 shows the logistic regressions made to evaluate the presence of a DRM at I50 codon.

**Table 5:** Logistic regression to evaluate the association of demographic and laboratory variables to the presence of I50LV mutations.

	Unadjusted			Adjusted		
	Odds ratio	p	95% CI	Odds ratio	p	95% CI
Age above 36 Years	3.72	0.000	1.93–7. 17	2.30	0.141	0.76–6.97
Male or female sex	0.96	0.828	0.66–1.40			
CD4 T count >200 cells/mm^3^	0.87	0.478	0.58–1.29			
CD4 T count >500 cells/mm^3^	1.55	0.041	1.02–2.36	1.19	0.644	0.56–2.53
Viral load <1.000 copies/ml	0.93	0.774	0.58–1.50			
Viral load >100.000 copies/ml	0.77	0.395	0.42–1.40			
Time on treatment >7 years	4.27	0.000	2.24–8.12	2.25	0.099	0.86–5.88
Number of regimens >2	4.25	0.000	2.26–8.00	1.01	0.992	0.36–2.85
NRTI mutation	18.23	0.000	5.77–57.65	19.54	0.004	2.61–146.10
NNRTI mutation	1.26	0.249	0.85–1.87			
Exposure to IDV, AMP, NFV	2.76	0.000	1.69–4.49	2.14	0.057	0.98–4.70
LPV treatment	1.01	0.969	0.62–1.64			
ATV treatment	4.67	0.000	2.67–8.18	4.35	0.000	1.99–9.51
DRV treatment	4.76	0.000	2.63–8.64	4.07	0.002	1.65–10.05
HIV-1 Polymerase subtype F	1.80	0.033	1.05–3.09	3.36	0.003	1.50–7.52

Logistic regression of variables at the genotyping collection associated to PI mutations. NRTI: nucleotide analog reverse transcriptase inhibitor; NNRTI: non-nucleoside reverse transcriptase inhibitor; IDV: Indinavir; SQV: Saquinavir; NFV: Nelfinavir; LPV: Lopinavir; ATV: Atazanavir; DRV: Darunavir.

As can be seen in Table 5, many demographic and laboratory data show some association to the presence of mutation at codon I50, but at adjusted analysis only the presence of NRTI mutations, exposure to any PI and subtype F remained significant.

## Discussion

Protease inhibitors (PI) have been used since late 1990s as part of the combined antiretroviral therapy. With the development of NNRTI class drugs, and more recently, integrase inhibitors, PI have been reserved mostly to second and subsequent therapy combinations for patients failing initial regimen [15–16].

Emergence of DRM to PIs is not as common as to NRTI and NNRTI class. The narrow drug concentration range that is both low enough to allow viral replication but has a sufficient drug level to provide selective pressure is considered a major issue for the limited detection of resistance mutations in patients failing boosted PIs. Albeit mutations outside of protease region have also been suggested as a mechanism for resistance, no conclusive resistance pattern has been identified [17].

To access the degree of protease resistance and predictors of PI-DRM, we evaluated a large data set of HIV-1 *pol* sequences and associated demographic and laboratory information from patients exposed to at least one PI containing regimen. In about one out of four patients at least one PI-DRM was observed (27.5%, 466/1696). The number of PI-DRM identified is higher than that reported for first or second line failures [18], but many (47.8%, 383/801) patients in our study used three or more regimens, many exposed to two or more PIs. Although we did not document the time on failure, some patients may have remained viremic for some time before genotype testing, increasing the chance of resistance emergence. There was a clear association to number of PI used and the number of PI-DRM.

Lopinavir was used by most cases and the most prevalent mutations, at codons 46, 54 and 82, have also been commonly found in other studies that include patients failing Lopinavir based second line regimens in LMICs settings [19–20]. The use of lopinavir and the other PIs currently in use, as well as older drugs (indinavir, saquinavir and nelfinavir), are all associated to the presence of mutations, but incomplete actual usage precluded further evaluation. As darunavir was used at the time mostly in salvage therapy of patients failing other PI regimens, the association of this drug to the presence of PI-DRM must take this fact into account.

Moreover, all PI use was considered, and as drugs were used sequentially in half of the patients, we included specific PI in the regression analyses not to determine its role in resistance but rather to evaluate if any particular protease inhibitor impacted the associations observed. Presence of a NRTI, but not NNRTI mutation, showed the strongest association to the detection of a PI-DRM, with only 4.7% of cases with PI-DRM not having a NRTI-DRM. Although the association may derive from the fact that the presence of mutations to other classes used in an ARV combination can favor the emergence of PI resistance, NRTI mutations may serve as a proxy for adequate adherence treatment, a potential marker of enough drug selective pressure at some point to allow PI-DRM evolution.

Subtype F but not subtype C was associated to the presence of PI-DRM in adjusted analysis. Consistent to this more recent introduction, subtype C had much lower rates of resistance in most codons, and in all adjusted analysis these rates seems to dependent to other variables, as shorter time of ARV treatment and lower number of regimens. As a consequence, many PI-DRM were not identified among HIV-1 C infected individuals. One exception is at codon I50. Rates for these codons are comparable to other subtypes and even significantly higher than HIV-1 B in some sub-analysis, as for patients with at least one PI, but this needs further evaluation. Subtype F, on the contrary, tended to show marginally higher rates of mutations in most unadjusted analysis, and subtype F was significantly associated in adjusted analysis to the presence of PI-DRM, to lower DRV and ATV GSS as well to the presence of mutations at codon I50. Though this has not been reported before, it is in line with Stanford db “Mutation Prevalence According to Subtype and Treatment” tool (https://hivdb.stanford.edu/cgi-bin/MutPrevBy SubtypeRx.cgi), that registers higher rates of I50 mutations for subtype F treated patients in their dataset.

We evaluated nucleotide composition of the codon 50 and flanking region to look for some potential molecular signatures that could facilitate resistance evolution, but we could not identify any. Potential epistatic influences from other regions of the HIV-1 F genome were not evaluated and cannot be ruled out. These findings assume more relevance with recent description of a lower response of subtype F to integrase inhibitors based regimens [21].

Associations of subtype and specific mutations have already been described, as the NNRTI mutation V106M, more common in subtype C possibly due to the need of only one nucleotide mutation to change the wild amino acid to a resistant one [22]. K65R mutations, conferring resistance to the NRTI drug tenofovir, have been associated to subtype C [23] and the PI-DRM V82M seems more frequent in subtype G [24]. Studies in Spain [21], suggest lower rates of virological suppression in patients using different regimens but polymorphisms common to subtype F were not associated to virological response. The authors suggest that a high replication capacity could play a role. This would lead to higher viremia among subtype F patients observed in their study. In our population, the viral load at failure is higher than 100,000 c/mL in 20% of subtype F compared to 15% of subtype B (p = 0.1), but this may not represent the viremia observed before use of antiretroviral drugs, and the patients we analyzed were all failing treatment. Interestingly, analysis of the subtype F circulating in the northwest of Spain have suggested that it originated from Brazil [21,25].

There are many limitations to our study. It is a retrospective evaluation where part of the information was missing, as information on total treatment time and detailed PI exposure, available for some patients only. We do not have data on the actual use boosted PI, although it has been recommended in treatment guidelines. The lower rates of PI-DRM among cases with less time on treatment may be related to the protective effect of boosted PI to the emergence of resistance or simply the increased chance of developing a PI-DRM with longer time on PI therapy.

Moreover, as some PI, as darunavir, were reserved for salvage therapy, including in regimens for patients failing PI regimens due to resistance, its association to PI resistance is not unexpected. Our study cannot evaluate the association of individual PI and resistance. We included all sequences that had a minimum of demographic and laboratory data associated to it, and therefore cases not having access to genotyping, or not amplifying in the assays used, were not accounted for. On the other hand, in the study period we received samples for genotyping test from most of the services in the State that provide specialized care to people living with HIV, and in a way it represents the actual population of patients failing an ARV regimen in the region.

In the adjusted analysis, inclusion in the model of variables with information available for only some cases implies a smaller sample size, but results are in line with the logistic performed using only data available for all cases, as age, DRM, subtype and sex. On the other hand these analyses are based in a large sequence dataset, with information generated for subtype and resistance using known and validated internet resources as Stanford database, NCBI and Rega genotyping tool. Adherence and drug levels are necessary to the emergence of resistance and it is an important issue when one tries to evaluate potential associations to the presence of DRM or a compromised GSS. The use of NRTI mutation may serve as a proxy for adherence at some point, but proper adherence information is a major limitation to our analyses.

## Conclusion

Major resistance mutations to PI, although not as high as to NRTI or NNRTI antiretroviral class is present in 27.5% of PI exposed patients, reducing the susceptibility of available PI full activity, including darunavir, with only 57% fully susceptible among those patients with PI mutations. Demographic data, as longer time on treatment are associated to PI-DRM, and the presence of a NRTI mutation is observed in almost all patients with PI-DRM and may provide a proxy for adequate adherence to allow protease resistance emergence. The more recently introduced subtype C in the area is associated to lower number of regimens and time on treatment, which may account for the observed lower rates of resistance. On the contrary, subtype F shows worse rates both to presence of resistance as well as lower GSS to atazanavir and darunavir. Mutation at codon I50 seems to be more frequent among non-B subtypes evaluated, but we did not identify any signature at or near the codon that could justify this findings.

## References

[1] PATON Nicholas I. et al Assessment of Second-Line Antiretroviral Regimens for HIV Therapy in Africa. New England Journal Of Medicine, [s.l.], v. 371, n. 3, p.234–247, 17 7 2014 New England Journal of Medicine (NEJM/MMS). 10.1056/nejmoa1311274. 25014688

[2] A BOYD Mark et al Baseline HIV-1 resistance, virological outcomes, and emergent resistance in the SECOND-LINE trial: an exploratory analysis. The Lancet Hiv, [s.l.], v. 2, n. 2, p.42–51, 2 2015 Elsevier BV. 10.1016/s2352-3018(14)00061-7. 26424460

[3] MATSUDA Elaine Monteiro et al High Prevalence of Drug Resistance Mutations Among Patients Failing First-Line Antiretroviral Therapy and Predictors of Virological Response 24 Weeks After Switch to Second-Line Therapy in São Paulo State, Brazil. Aids Research And Human Retroviruses, [s.l.], v. 34, n. 2, p.156–164, feb. 2018. Mary Ann Liebert Inc. 10.1089/aid.2017.0052. 28969448

[4] LÓPEZ-CORTÉS Luis F. et al Effectiveness of Ritonavir-Boosted Protease Inhibitor Monotherapy in Clinical Practice Even with Previous Virological Failures to Protease Inhibitor-Based Regimens. Plos One, [s.l.], v. 11, n. 2, p.1–12, 12 2 2016 Public Library of Science (PLoS). 10.1371/journal.pone.0148924. PMC4752289 26872331

[5] UNAIDS. Infográficos—Brasil. 2017. Disponível em: <https://unaids.org.br/wp-content/uploads/2015/06/20170720_DaDOS_unaids_Brasil.pdf>. Acessoem: 14 feb. 2019

[6] BRÍGIDO L. F. M. et al Southern Brazil HIV Type 1 C Expansion into the State of São Paulo, Brazil. Aids Research And Human Retroviruses, [s.l.], v. 27, n. 3, p.339–344, 3 2011 Mary Ann Liebert Inc. 10.1089/aid.2010.0157. 20950149

[7] COELHO Luana Portes Ozorio et al Prevalence of HIV-1 transmitted drug resistance and viral suppression among recently diagnosed adults in São Paulo, Brazil. Archives Of Virology, [s.l.], p.1–8, 20 12 2018 Springer Nature. 10.1007/s00705-018-04122-8 30569276

[8] HEMELAAR Joris et al Global and regional molecular epidemiology of HIV-1, 1990–2015: a systematic review, global survey, and trend analysis. The Lancet Infectious Diseases, [s.l.], v. 19, n. 2, p.143–155, 2 2019 Elsevier BV. 10.1016/s1473-3099(18)30647-9 30509777

[9] THOMSON Michael M. et al Rapid Expansion of a HIV-1 Subtype F Cluster of Recent Origin Among Men Who Have Sex With Men in Galicia, Spain. Jaids Journal Of Acquired Immune Deficiency Syndromes, [s.l.], v. 59, n. 3, p.49–51, 3 2012 Ovid Technologies (Wolters Kluwer Health). 10.1097/qai.0b013e3182400fc4. 22327248

[10] DELGADO Elena et al Phylogeny and Phylogeography of a Recent HIV-1 Subtype F Outbreak among Men Who Have Sex with Men in Spain Deriving from a Cluster with a Wide Geographic Circulation in Western Europe. Plos One, [s.l.], v. 10, n. 11, p.1–8, 24 11 2015 Public Library of Science (PLoS). 10.1371/journal.pone.0143325. PMC4658047 26599410

[11] RHEE Soo-yon et al HIV-1 *pol* mutation frequency by subtype and treatment experience: extension of the HIVseq program to seven non-B subtypes. Aids, [s.l.], v. 20, n. 5, p.643–651, 3 2006 Ovid Technologies (Wolters Kluwer Health). 10.1097/01.aids.0000216363.36786.2b. 16514293 PMC2551321

[12] ORTIZ Roberto et al Efficacy and safety of once-daily darunavir/ritonavir versus lopinavir/ritonavir in treatment-naive HIV-1-infected patients at week 48. Aids, [s.l.], v. 22, n. 12, p.1389–1397, 7 2008 Ovid Technologies (Wolters Kluwer Health). 10.1097/qad.0b013e32830285fb. 18614861

[13] MOLINA Jean-michel et al Once-Daily Atazanavir/Ritonavir Compared With Twice-Daily Lopinavir/Ritonavir, Each in Combination With Tenofovir and Emtricitabine, for Management of Antiretroviral-Naive HIV-1-Infected Patients: 96-Week Efficacy and Safety Results of the CASTLE Study. Jaids Journal Of Acquired Immune Deficiency Syndromes, [s.l.], v. 53, n. 3, p.323–332, 3 2010 Ovid Technologies (Wolters Kluwer Health). 10.1097/qai.0b013e3181c990bf 20032785

[14] GUIMARÃES, Paula Morena de Souza et al Transmitted Drug Resistance Among Recently Diagnosed Adults and Children in São Paulo, Brazil. Aids Research And Human Retroviruses, São Paulo, v. 31, n. 12, p.1219–1224, dec. 2015 Mary Ann Liebert Inc. 10.1089/aid.2014.0354. 25826640

[15] RYOM L et al Highlights of the 2017 European AIDS Clinical Society (EACS) Guidelines for the treatment of adult HIV-positive persons version 9.0. Hiv Medicine, [s.l.], v. 19, n. 5, p.309–315, 1 3 2018 Wiley. 10.1111/hiv.12600. 29493093 PMC5947127

[16] UNITED STATES DEPARTMENT OF HEALTH AND HUMAN SERVICES. Guidelines for the Use of Antiretroviral Agents in Adults and Adolescents Living with HIV. 2018 Disponível em: <https://aidsinfo.nih.gov/guidelines/html/1/adult-and-adolescent-arv/11/what-to-start>. Access: 10 jan. 2019.

[17] CLUTTER Dana S. et al HIV-1 drug resistance and resistance testing. Infection, Genetics And Evolution, [s.l.], v. 46, p.292–307, dec. 2016. Elsevier BV. 10.1016/j.meegid.2016.08.031. PMC5136505 27587334

[18] SHAFER Robert W. Human Immunodeficiency Virus Type 1 Drug Resistance Mutations Update. The Journal Of Infectious Diseases, [s.l.], v. 216, n. 9, p.843–846, 15 9 2017 Oxford University Press (OUP). 10.1093/infdis/jix398. PMC5853262 28968669

[19] A THOMPSON Jennifer et al Evolution of Protease Inhibitor Resistance in Human Immunodeficiency Virus Type 1 Infected Patients Failing Protease Inhibitor Monotherapy as Second-line Therapy in Low-income Countries: An Observational Analysis Within the EARNEST Randomized Trial. Clinical Infectious Diseases, [s.l.], v. 68, n. 7, p.1184–1192, 28 7 2018 Oxford University Press (OUP). 10.1093/cid/ciy589. 30060027

[20] FILY F. et al HIV-1 drug resistance testing at second-line regimen failure in Arua, Uganda: avoiding unnecessary switch to an empiric third-line. Tropical Medicine & International Health, [s.l.], v. 23, n. 10, p.1075–1083, 10 2018 Wiley. 10.1111/tmi.13131. 30058269

[21] CID-SILVA Purificación et al Initial treatment response among HIV subtype F infected patients who started antiretroviral therapy based on integrase inhibitors. Aids, [s.l.], v. 32, n. 1, p.121–125, 1 2018 Ovid Technologies (Wolters Kluwer Health). 10.1097/qad.0000000000001679. 29112068

[22] GROSSMAN et al Genetic variation at NNRTI resistance-associated positions in patients infected with HIV-1 subtype C. Aids, London, v. 18, n. 6, p.909–915, 4 2004 10.1097/00002030-200404090-00008 15060438

[23] COUTSINOS D. et al Template Usage Is Responsible for the Preferential Acquisition of the K65R Reverse Transcriptase Mutation in Subtype C Variants of Human Immunodeficiency Virus Type 1. Journal Of Virology, [s.l.], v. 83, n. 4, p.2029–2033, 10 12 2008 American Society for Microbiology. 10.1128/jvi.01349-08. 19073730 PMC2643749

[24] PALMA A. C. et al HIV-1 protease mutation 82M contributes to phenotypic resistance to protease inhibitors in subtype G. Journal Of Antimicrobial Chemotherapy, [s.l.], v. 67, n. 5, p.1075–1079, 13 2 2012 Oxford University Press (OUP). 10.1093/jac/dks010. 22331593

[25] PARASKEVIS Dimitrios et al Molecular characterization of HIV-1 infection in Northwest Spain (2009–2013): Investigation of the subtype F outbreak. Infection, Genetics And Evolution, [s.l.], v. 30, p.96–101, 3 2015 Elsevier BV. 10.1016/j.meegid.2014.12.012. 25527396

